# Effects of black soldier fly larvae as protein or fat sources on apparent nutrient digestibility, fecal microbiota, and metabolic profiles in beagle dogs

**DOI:** 10.3389/fmicb.2022.1044986

**Published:** 2022-11-25

**Authors:** Shiyan Jian, Limeng Zhang, Ning Ding, Kang Yang, Zhongquan Xin, Minhua Hu, Zhidong Zhou, Zhihong Zhao, Baichuan Deng, Jinping Deng

**Affiliations:** ^1^Maoming Branch, Guangdong Laboratory for Lingnan Modern Agriculture, Guangdong Provincial Key Laboratory of Animal Nutrition Control, National Engineering Laboratory for Pollution Control and Waste Utilization in Livestock and Poultry Production, National Engineering Research Center for Breeding Swine Industry, College of Animal Science, South China Agricultural University, Guangzhou, China; ^2^Guangzhou Qingke Biotechnology Co., Ltd., Guangzhou, Guangdong, China; ^3^Guangzhou Customs Technology Center, Guangzhou, Guangdong, China; ^4^Guangzhou General Pharmaceutical Research Institute Co., Ltd. (National Canine Laboratory Animal Resources Center), Guangzhou, Guangdong, China

**Keywords:** black soldier fly larvae, pet food, beagle dog, protein and fat, apparent nutrient digestibility, microbiota, metabolomics

## Abstract

Black soldier fly (*Hermetia illucens*) larvae (BSFL) act as a biological system converting organic waste into protein and fat with great potential application as pet food. To evaluate the feasibility of BSFL as a protein and fat source, 20 healthy beagle dogs were fed three dietary treatments for 65 days, including (1) a basal diet group (CON group), (2) a basal diet that replaced 20% chicken meal with defatted black soldier fly larvae protein group (DBP group), and (3) a basal diet that replaced 8% mixed oil with black soldier fly larvae fat group (BF group). This study demonstrated that the serum biochemical parameters among the three groups were within the normal range. No difference (*p* > 0.05) was observed in body weight, body condition score, or antioxidant capacity among the three groups. The mean IFN-γ level in the BF group was lower than that in the CON group, but there was no significant difference (*p* > 0.05). Compared with the CON group, the DBP group had decreasing (*p* < 0.05) apparent crude protein and organic matter digestibility. Furthermore, the DBP group had decreasing (*p* < 0.05) fecal propionate, butyrate, total short-chain fatty acids (SCFAs), isobutyrate, isovalerate, and total branched-chain fatty acids (BCFAs) and increased (*p* < 0.05) fecal pH. Nevertheless, there was no difference (*p* > 0.05) in SCFAs or BCFAs between the CON and BF groups. The fecal microbiota revealed that *Lachnoclostridium*, *Clostridioides*, *Blautia*, and *Enterococcus* were significantly enriched in the DBP group, and *Terrisporobacter* and *Ralstonia* were significantly enriched in the BF group. The fecal metabolome showed that the DBP group significantly influenced 18 metabolic pathways. Integrating biological and statistical correlation analysis on differential fecal microbiota and metabolites between the CON and DBP groups found that *Lachnoclostridium*, *Clostridioides*, and *Enterococcus* were positively associated with biotin. In addition, *Lachnoclostridium*, *Clostridioides*, *Blautia*, and *Enterococcus* were positively associated with niacinamide, phenylalanine acid, fumaric acid, and citrulline and negatively associated with cadavrine, putrescine, saccharopine, and butyrate. In all, 20% DBP restrained the apparent CP and OM digestibility, thereby affecting hindgut microbial metabolism. In contrast, 8% BF in the dog diet showed no adverse effects on body condition, apparent nutrient digestibility, fecal microbiota, or metabolic profiles. Our findings are conducive to opening a new avenue for the exploitation of DBP and BF as protein and fat resources in dog food.

## Introduction

With the increase in the population of humans and animals, the shortages of protein and fat, mainly referring to fishmeal (Dawood and Koshio, 2020; Luthada-Raswiswi et al., 2021) and soybean (Herrera et al., 2022), is an urgent problem. Apart from traditional economic animals, the amount of pets, mainly dogs and cats, is also in a booming development stage, and the demand for pet food has further increased (De Marchi et al., 2018). In addition, meats, such as chicken, swine, bovine, fish, and exotic meats, and meat and bone byproducts are the major protein sources of dog food, which also fail to meet the increasing demands of the pet industry (Swanson et al., 2013). It is well known that dogs are omnivorous animals, cats are carnivorous animals, and the levels of animal protein and fat are high in their diets, which has promoted the development of new high-quality and sustainable protein and fat sources for pet food (Bosch et al., 2016). Hence, it is necessary to develop novel protein and fat sources.

Insects, which have been successfully introduced in animal diets (poultry, swine, rabbits, fish, and pets) in recent years, have bright prospects as alternate protein and fat sources (Benzertiha et al., 2020). Protein from insects shows high biological value, and fat may replace palm (i.e., kernel) fat and hence contribute to the conservation of tropical forests (Müller et al., 2017). The three most evaluated insects, black soldier fly (*Hermetia illucens*) larvae (BSFL), mealworm, and adult cricket, have high protein contents (dry matter basis) and are similar to soybean meal and meat meal (Valdés et al., 2022). Moreover, these insects are rich in essential amino acids (Bosch et al., 2014), such as aspartic acid, glutamic acid, valine, leucine, and alanine, which are similar to those of animals (Churchward-Venne et al., 2017) and have a high digestibility (76–98%; Ramos-Elorduy et al., 1997). Particularly, among these insects, BSFL is a highly investigated insect due to its strong fecundity, high conversion rate, high nutrition, low cost, and easy management (Yildirim Aksoy et al., 2020), and it is most commonly used in pet food (Valdés et al., 2022). In detail, BSFL promote environmental sustainability by converting a vast amount of low-value organic wastes, such as vegetables, fruits, and garbage, into protein and fat (Kim et al., 2011; Kelemu et al., 2015), leaving behind a compost-like residue that can be used as a soil conditioner (Somroo et al., 2019). Moreover, BSFL is rich in fatty acids (Tschirner and Simon, 2015; Ewald et al., 2020), including lauric acid, palmitic acid, oleic acid, linoleic acid, and linolenic acid, and contains abundant amino acids (Do et al., 2020), such as arginine, histidine, isoleucine, leucine, and lysine, which suggests that BSFL is an excellent raw feed material. Early studies have demonstrated that BSFL showed accelerative effects on the growth performance and nutrient digestibility of finishing pigs (Hong and Kim, 2022), was a suitable substitute for soybean meal in the diet of poultry (Józefiak et al., 2016; Mwaniki et al., 2018; Secci et al., 2018), and acted as a complementary protein source in dog diets with characteristics comparable to fish meal (Freel et al., 2021). Recently, a similar study was conducted to assess the digestibility and safety, including dry matter, protein, fat, energy, and hematology parameters of BSFL and BSFL fat, in beagle dogs, and some referential results were obtained (Freel et al., 2021). However, the investigation of BSFL as a protein or fat material in pets is highly limited and not profound enough to explore the relationship between intestinal health (Bruno et al., 2019) and metabolic variation.

Thus, the purpose of this study was to explore the effects of protein and fat isolated from BSFL on apparent nutrient digestibility, serum biochemistry, antioxidant and anti-inflammatory properties, and fecal SCFAs in dogs. In addition, we further detected fecal microbiota and metabolic profiles through 16S rRNA amplicon sequencing and ultra-performance liquid chromatography-Orbitrap-tandem mass spectrometry (UPLC-Orbitrap-MS/MS) and mined the potential relationships between microbiota and metabolites. This study is conducive to providing a new understanding of the exploitation of defatted black soldier fly larvae protein (DBP) and black soldier fly larvae (BF) as protein and fat resources.

## Materials and methods

### Preparation of defatted black soldier fly larvae protein and black soldier fly larvae fat

The DBP and BF were purchased from Guangzhou Unique Biotechnology Co., Ltd., and BSFL aged 10–12 days were reared with kitchen waste under constant temperature and humidity (28°C, 80%). The BSFL were killed by the refrigeration technique by keeping the temperature under − 20°C for 24 h and then oven drying under 65°C for 24 h. The dried BSFL were boiled in a steamer for 20–30 min and then stir-fried in a wok for 10–20 min. Afterward, the stir-fried BSFL was finely pulverized and prepared using n-hexane as the solvent in the press, and the leach solution was collected as the BF. Next, the DBP was extracted by alkaline solution and acid precipitation, dialysis desalting, and lyophilization.

### Animals, diets, and experimental design

The animal experimental procedures mentioned in this study were reviewed and approved by the Experimental Animal Ethics Committee of South China Agricultural University (approval code 2021E028).

After a month of adaptation, 20 beagle dogs [mean age: 10 months; mean body weight (BW): 12.67 ± 1.48 kg; mean body condition score (BCS): 5.84 ± 0.71] were randomly allotted to three dietary treatments according to their gender and BW. The dietary treatments included the following: (1) a basal diet group (CON group; *n* = 6, 2 male and 4 female); (2) a basal diet that replaced 20% chicken meal with 20% defatted black soldier fly larvae protein group (DBP group; *n* = 7, 3 male and 4 female); and (3) a basal diet that replaced 8% mixed oil with 8% black soldier fly larvae fat group (BF group; *n* = 7, 3 male and 4 female). These extruded diets exceeded the nutrient requirements of adult dogs recommended by the Association of American Feed Control Officials (AFFCO; AAFCO Official Publication, 2022). Table 1 shows the ingredients and nutrient levels of the experimental diets, and Supplementary Table 1 presents the proximate analysis data of chicken meal and DBP. The three kinds of dog food were made at Guangzhou Qingke Biotechnology Co., Ltd., and the experimental period lasted for 65 days, including a 5-day preliminary trial period and a 60-day formal trial period.

**Table 1:** Ingredients and nutrient levels of the experimental diets (as-fed basis, %).

Items	CON	DBP	BF
Ingredients	as-fed basis, %		
Corn	25.00	25.00	25.00
Sweet potato flour	12.00	12.00	12.00
Wheat flour	10.00	10.00	10.00
Corn gluten meal	5.00	5.00	5. 00
Beet pulp	2.50	2.50	2.50
Duck meal	8.00	8.00	8.00
Fish meal	2.50	2.50	2.50
Meat and bone meal	3.00	3.00	3.00
Calcium bicarbonate	1.00	1.00	1.00
Solid flavor enhancer	2.00	2.00	2.00
Vitamin and mineral premix^1^	1.00	1.00	1.00
Chicken meal	20.00	0	20.00
Mixed oil^2^	8.00	8.00	0
Defatted black soldier fly protein	0	20.00	0
Black solider fly fat	0	0	8.00
Nutrient levels^3^			
DM (%)	91.20	91.29	92.66
OM (%)	93.68	92.49	93.25
CP (%)	32.72	30.21	32.55
EE (%)	14.30	16.15	15.99
GE (kcal/kg)	4759.86	4560.91	4598.08
Calcium (%)	0.57	0.53	0.50
Phosphorus (%)	0.46	0.42	0.41
Calcium and phosphorus ratio	1.24	1.26	1.22

CON: basal diet group; DBP: defatted black soldier fly larvae protein group; BF: black soldier fly larvae fat group; DM: dry matter; OM: organic matter; CP: crude protein; EE: ether extract; GE: gross energy.

1 Vitamin and mineral premix provided the following per kilogram of diet: vitamin A, 2,260,000 IU; vitamin D3, 50,000 IU; vitamin E, 5,400 mg; vitamin K3, 10 mg; vitamin B1 (thiamine), 1,680 mg; vitamin B2 (riboflavin), 740 mg; vitamin B6, 840 mg; vitamin B12, 3 mg; niacin, 9,800 mg; calcium pantothenate 948 mg; biotin, 11 mg; folacin, 90 mg; choline chloride, 264,180 mg; Fe, 8,000 mg; Cu, 1,500 mg; Mn, 780 mg; Zn, 7,520 mg; I, 180 mg; Se, 30 mg.

2 Mixed oil contained 2% fish oil and 6% chicken oil.

3 Measured values in dry matter basis.

All dogs were housed individually in custom-made stainless steel metabolism cages (1.2 × 1.0 × 1.1 m kennels) under a constant temperature and humidity (23°C, 70%) with a 12 h light/dark cycle. A restricted diet of 130 g per dog was offered at each of the two daily meals at 8:00 am and 5:00 pm. All dogs were dewormed and vaccinated before the experiment, and no drugs were used throughout the entire experiment. All dogs were always given fresh water and toys and socialized with humans at least once a day. Final BW and BCS (Cline et al., 2021) were performed on day 65 before the morning feeding.

### Diets, feces collection and analysis

Three diet samples (100 g) were collected when each bag of the three kinds of dog food was opened throughout the experimental period. Whole feces were collected, and 10% HCl was added to the nitrogen fixation on days 62–65. Diet and feces samples were stored at −20°C, oven-dried at 65°C for 48 h and finely ground to pass through a 1-mm mesh screen for subsequent analysis. The dry matter (DM) and organic matter (OM) contents of the diet and feces samples were determined according to the methods of the Association of Official Analytical Chemists (AOAC, 2000; Hortwitz and Latimer, 2007). Based on AOAC, crude protein (CP), ether extract (EE), and gross energy (GE) were determined with a semiautomatic Kjeldahl apparatus (VAPODEST 200, C. Gerhardt GmbH & Co. KG, Germany), fatty analyzer (FT640, Grand Analytical Instrument Co., Ltd., Guangzhou, China), and oxygen bomb calorimeter [IKA C 200, IKA (Guangzhou) Instrument Equipment Co., Ltd., Guangzhou, China], respectively. Finally, the apparent digestibility of nutrients was calculated by referencing the following formula: Apparent nutrient digestibility (%) = (Nutrient intake – Nutrient in feces)/Nutrient intake*100 (g/d, DM basis).

### Serum sample collection and analysis

On day 65, fasting blood was collected and placed for 30 min and centrifuged at 1,811 × *g* at room temperature for 15 min. Finally, aliquots for serum biochemical, antioxidant, inflammatory parameters and metabolomics analysis were snap-frozen in liquid N_2_ and stored at −80°C until analysis. The serum biochemical parameters, including albumin (ALB), total protein (TP), globulin (GLO), albumin/globulin (ALB/GLO), aspartate aminotransferase (AST), alanine transaminase (ALT), amylase (AMY), creatine kinase (CK), creatinine (CRE), urea nitrogen (BUN), glucose (GLU), calcium (Ca), and inorganic phosphorus (IP), were detected using an automatic biochemical analyzer (SMT-120VP, Chengdu Seamaty Technology Co., Ltd., Chengdu, China). According to the manufacturer’s protocol of commercial kits (Nanjing Jiancheng Bioengineering Institute, Nanjing, China), the serum contents of total antioxidant capacity (T-AOC), glutathione peroxidase (GSH-Px), malondialdehyde (MDA), catalase (CAT), and superoxide dismutase (SOD) were detected. Serum tumor necrosis factor-α (TNF-α), interferon-γ (IFN-γ), interleukin 6 (IL-6), IL-8, IL-10, IL-1β, immunoglobulin A (IgA), IgG, and IgM were measured using canine enzyme-linked immunosorbent assay (ELISA) kits (MEIMIAN, Jiangsu Meimian Industrial Co., Ltd., Jiangsu, China).

### Fecal 16S rRNA high-throughput sequencing analysis

On day 65, fresh fecal samples of each dog were collected within 15 min of defecation. A total of 0.5 g of each fecal sample was taken for the extraction of total fecal DNA using the CTAB method according to the manufacturer’s instructions. Determination of DNA concentration and purity using a NanoDrop2000. The 16S rRNA genes of V3–V4 were amplified with the barcode using the primers 515F (5′-GTGYCAGCMGCCGCGGTAA-3′) and 805R (5′-GGACTACHVGGGTWCTAAT-3′). PCRs were carried out with approximately 10 ng of template DNA and 15 μl of Phusion^®^ High-Fidelity PCR Master Mix (New England Biolabs) with 2 μM forward and reverse primers. The cycling parameters consisted of 98°C for 30 s, followed by 32 cycles of denaturation at 98°C for 10 s, annealing at 54°C for 30 s, and elongation at 72°C for 45 s, followed by 72°C for 10 min. PCR amplification products were detected by 2% agarose gel electrophoresis, and the target fragments were recovered using the AxyPrep PCR Cleanup Kit. The purified PCR products were quantified by a Quant-iT PicoGreen dsDNA Assay Kit on a Qbit fluorescence quantitative system, and the qualified library concentration was above 2 nM. The qualified libraries (index sequence could not be repeated) were gradient diluted, mixed according to the required amount of sequencing in proportion, and denatured by NaOH into a single chain for on-machine sequencing. A NovaSeq 6000 sequence analyzer was used for 2 × 250 bp double-end sequencing, and the corresponding reagent was a NovaSeq 6000 SP Reagent Kit (500 cycles).

After the on-board sequencing was completed, we obtained the original off-board data RawData, used overlap to splice the dual-end data, and performed quality control chimaism filtering to obtain the high-quality CleanData. DADA2 (Divisive Amplicon Denoising Algorithm; Callahan et al., 2016) no longer clusters in sequence similarity but instead clusters by dereplication (Dereplication, equivalent to clustering with 100% similarity) to obtain representative sequences with single base accuracy, which greatly improves data accuracy and species resolution. The core of DADA2 was denoised, and then amplicon sequence variants (ASVs) were constructed (Blaxter et al., 2005) to obtain the final ASV feature table and feature sequence and to further conduct diversity analysis, species classification annotation, difference analysis, etc. Based on these output-normalized data, subsequent analyses of alpha diversity and beta diversity were performed. Alpha diversity, including Observed_species, Chao1, Shannon, Simpson, and Pielou_e, was applied to analyze species diversity and richness. All these indices were calculated using QIIME 2 (Version QIIME2-202006). We calculated the linear discriminant analysis (LDA) effect size (LEfSe) using LEfSe software^1^ with the default setting of LDA score ≥ 3.

### Fecal fermentation metabolite analysis

Fresh fecal samples from each dog were collected within 15 min of defecation at the end of the 65-day intervention, and pH was measured immediately after mixing the 10% fecal suspension with ultrapure water using a portable pH meter (Starter 3,100, Ohaus Instruments Co., Ltd., Shanghai, China). Fecal samples were snap-frozen in liquid N_2_ and stored at −80°C for further analysis. The fecal short-chain fatty acids (SCFAs) and branched-chain fatty acids (BCFAs) were measured by gas chromatography–mass spectrometry (GC–MS; Shimadzu, Tokyo, Japan) with a DB-FFAP capillary column (30 m × 0.25 mm × 0.25 μm, Onlysci, China). The instrument parameters and sample processing procedures were performed according to Yang et al. (2021).

### Fecal and serum untargeted metabolomics analysis

#### Fecal untargeted metabolomics analysis

Frozen fecal samples were thawed at 4°C, and approximately 60 mg of sample was put into 2-ml round-bottom centrifuge tubes. Magnetic beads and 600 μl of methanol:water (1:1, *v*/*v*) were added to the centrifuge tubes for homogenization to extract the fecal metabolites. Ultrasonic crushing was performed at a low temperature for 10 min and placed at −20°C for 30 min. The samples were then centrifuged at 19,745 × *g* and 4°C for 15 min, and 200 μl of supernatant was dried in a vacuum centrifuge. Then, the samples were redissolved with 200 μl of methanol (chromatographic grade) water and vortexed for 2 min. After ice bath ultrasonication for 10 min at low temperature, the microcentrifuge tube was centrifuged again at 19,745 × *g* and 4°C for 15 min. The supernatant was placed in a sample bottle with a lined tube and stored at −80°C. Fecal untargeted metabolomic analysis was performed using the UPLC-Orbitrap-MS/MS system from Thermo Fisher Scientific (Q-Exactive Focus, United States).

#### Serum untargeted metabolomics processing

Frozen serum samples were thawed at 4°C and vortexed for 2 min. Then, 200 μl of each serum sample was added to 800 μl of methanol (chromatographic grade). Then, the mixed solution was sequentially vortexed for 2 min and centrifuged at 19745 × *g* and 4°C for 15 min (Eppendorf, Centrifuge 5,424, Germany), after which 800 μl of the supernatant was dried in a vacuum centrifuge and processed immediately. The detection procedure was similar to that for the fecal samples.

#### Ultra-performance liquid chromatography-Orbitrap-MS/MS analysis and metabolite profiling analysis

The UPLC-Orbitrap-MS/MS analysis method was described in a previous work (Xin et al., 2018). Briefly, the raw data were processed by Compound Discoverer 2.1 software (Thermo Fisher Scientific, USA) to produce a data matrix including retention time (RT), mass spectrometry (m/z), and peak intensity. Meanwhile, metabolic features with a relative standard deviation greater than 30% were excluded. Then, we searched the mzCloud and mzVault libraries to identify metabolites from these data.

Principal component analysis (PCA), orthogonal partial least squares discriminant analysis (OPLS-DA) and response permutation testing (RPT) were performed using SIMCA-P 14.1 software (Umetrics, Umea, Sweden). OPLS-DA was applied to better understand the different metabolic patterns, and RPT was conducted to examine the accuracy of the OPLS-DA models.

### MetOrigin analysis

MetOrigin is a web server analysis system that integrates microbiome and metabolome data by providing the quick identification of microbiota-related metabolites and their metabolic functions in metabolomics studies (Yu et al., 2022). We performed the origin analysis, function analysis, correlation analysis, and network summary using MetOrigin analysis. Functional analysis was performed to perform metabolic pathway enrichment analysis according to different categories of metabolites: metabolites belonging to the host, bacteria, or both. Correlation analysis was performed to determine the correlation between microbiota at different levels and metabolites by Spearman analysis. Network summary highlights the interactions with both biological and statistical signification. MetOrigin analysis is freely available at http://metorigin.met-bioinformatics.cn/.

### Statistical analysis

All data were analyzed by SPSS 26.0, graphical presentation was performed using GraphPad Prism 8.0 software, and the results were expressed as the mean ± standard error (mean ± SE). *p*-values were determined using an unpaired Student’s *t test* for comparisons between two groups. *p* < 0.05 and *p* < 0.10 indicated significant differences and tendencies, respectively. Furthermore, variable importance in the projection (VIP) was calculated in the OPLS-DA model. The metabolites with VIP > 1 and *p* < 0.05 were deemed differential metabolites. The KEGG database was applied to functionally annotate these differential metabolites, which were further mapped to the KEGG pathway database using MetaboAnalyst 5.0.^2^

## Results

### Effects of DBP and BF on BW, BCS, and apparent nutrient digestibility in dogs

The effects of DBP and BF on BW, BCS, and the apparent nutrient digestibility of dogs are shown in Table 2. At the end of the experiment, BW and BCS among the three groups showed no difference (*p* > 0.05). Compared to the CON group, the apparent CP and OM digestibility in the DBP group were significantly decreased (*p* < 0.05), while apparent DM, EE, and GE digestibility were not different (*p* > 0.05). All apparent nutrient digestibilities in the BF group showed no differences compared with the CON group (*p* > 0.05).

**Table 2:** Effects of DBP and BF on BW, BCS, and apparent nutrient digestibility in dogs.

Items^1^	CON	DBP	BF	*p*-Value (CON/DBP)	*p*-Value (CON/BF)
BW	13.70 ± 2.07	13.47 ± 2.02	13.14 ± 1.71	0.847	0.602
BCS	6.33 ± 1.25	6.21 ± 1.11	5.86 ± 0.56	0.860	0.381
DM (%)	82.2 ± 0.01	79.86 ± 0.03	78.91 ± 0.61	0.106	0.281
OM (% DM)	87.01 ± 0.20	83.93 ± 0.71	85.90 ± 0.98	0.046	0.203
CP (% DM)	82.46 ± 0.37	72.70 ± 0.85	79.64 ± 0.56	0.001	0.132
EE (% DM)	95.47 ± 0.81	95.95 ± 0.68	93.92 ± 0.51	0.226	0.254
GE (% DM)	87.80 ± 0.51	86.63 ± 0.02	86.01 ± 0.21	0.180	0.135

^1^CON: basal diet group; DBP: defatted black soldier fly larvae protein group; BF: black soldier fly larvae fat group; BW: body weight; BCS: body condition score; DM: dry matter; CP: crude protein; EE: ether extract; OM: organic matter; GE: gross energy.

### Effects of DBP and BF on serum biochemistry, antioxidant, and inflammatory parameters in dogs

The effects of DBP and BF on serum biochemistry, antioxidant, and inflammatory parameters are presented in Supplementary Table 2. Neither the DBP group nor the BF group affected the serum biochemical parameters ALB, GLO, ALB/GLO, AST, ALT, AMY, CK, CRE, BUN, GLU, Ca, and IP compared with the CON group (*p* > 0.05).

The effects of DBP and BF on serum antioxidant and inflammatory parameters are presented in Table 3. Neither the DBP group nor the BF group affected the serum antioxidant parameters GSH-Px, MDA, T-AOC, CAT, and SOD and serum inflammatory parameters TNF-α, IL-6, IL-8, IL-10, IL-1β, IgA, IgG, and IgM compared with the CON group (*p* > 0.05). However, the mean IFN-γ level in the BF group was lower than that in the CON group but showed no significant difference (*p* > 0.05).

**Table 3:** Effects of DBP and BF on serum biochemical parameters in dogs.

Items^1^	CON	DBP	BF	*P*-value (CON/DBP)	*P*-value (CON/BF)
GSH-Px (U)	922.17 ± 158.94	952.97 ± 234.50	991.57 ± 189.98	0.792	0.508
SOD (U/ml)	134.59 ± 12.70	132.82 ± 6.91	121.20 ± 15.34	0.756	0.113
T-AOC (mM)	0.542 ± 0.04	0.545 ± 0.06	0.550 ± 0.10	0.943	0.861
CAT (U/ml)	2.43 ± 1.00	2.36 ± 1.37	2.29 ± 1.13	0.911	0.809
MDA (nmol/ml)	7.44 ± 5.81	6.04 ± 5.40	4.25 ± 2.12	0.646	0.593
TNF-α (ng/l)	149.82 ± 17.27	158.77 ± 18.28	159.84 ± 9.62	0.384	0.244
IFN-γ (ng/l)	39.16 ± 2.44	40.06 ± 3.62	36.45 ± 2.85	0.605	0.092^#^
IL-6 (g/l)	268.10 ± 32.42	246.46 ± 22.73	268.61 ± 25.82	0.204	0.976
IL-8 (ng/l)	90.42 ± 8.19	94.69 ± 12.30	95.32 ± 9.11	0.472	0.329
IL-10 (ng/l)	47.48 ± 3.41	47.32 ± 4.22	48.06 ± 1.95	0.943	0.721
IL-1β (ng/l)	78.89 ± 7.63	79.13 ± 7.18	74.68 ± 7.75	0.954	0.346
IgA (μg/ml)	9.54 ± 1.15	9.69 ± 0.75	9.09 ± 1.03	0.795	0.473
IgG (ng/l)	46.88 ± 4.07	51.66 ± 2.47	50.18 ± 2.93	0.461	0.133
IgM (ng/l)	5.10 ± 0.42	5.02 ± 0.47	4.92 ± 0.57	0.748	0.525

^1^CON: basal diet group; DBP: defatted black soldier fly larvae protein group; BF: black soldier fly larvae fat group; GSH-Px: glutathione peroxidase; MDA: malondialdehyde; T-AOC: total antioxidant capacity; CAT: catalase; SOD: superoxide dismutase; IFN-γ: interferon-γ; IL-6: interleukin 6; IL-8: interleukin 8; IL-10: interleukin 10; IL-1β: interleukin 1β; IgA: immunoglobulin A; IgG: immunoglobulin G; IgM: immunoglobulin M; TNF-α: tumour necrosis factor-α. The symbol (^#^) indicates *p* < 0.10 calculated by Student’s *t*-test.

### Effects of DBP and BF on serum metabolomics in dogs

**Figure 1:**
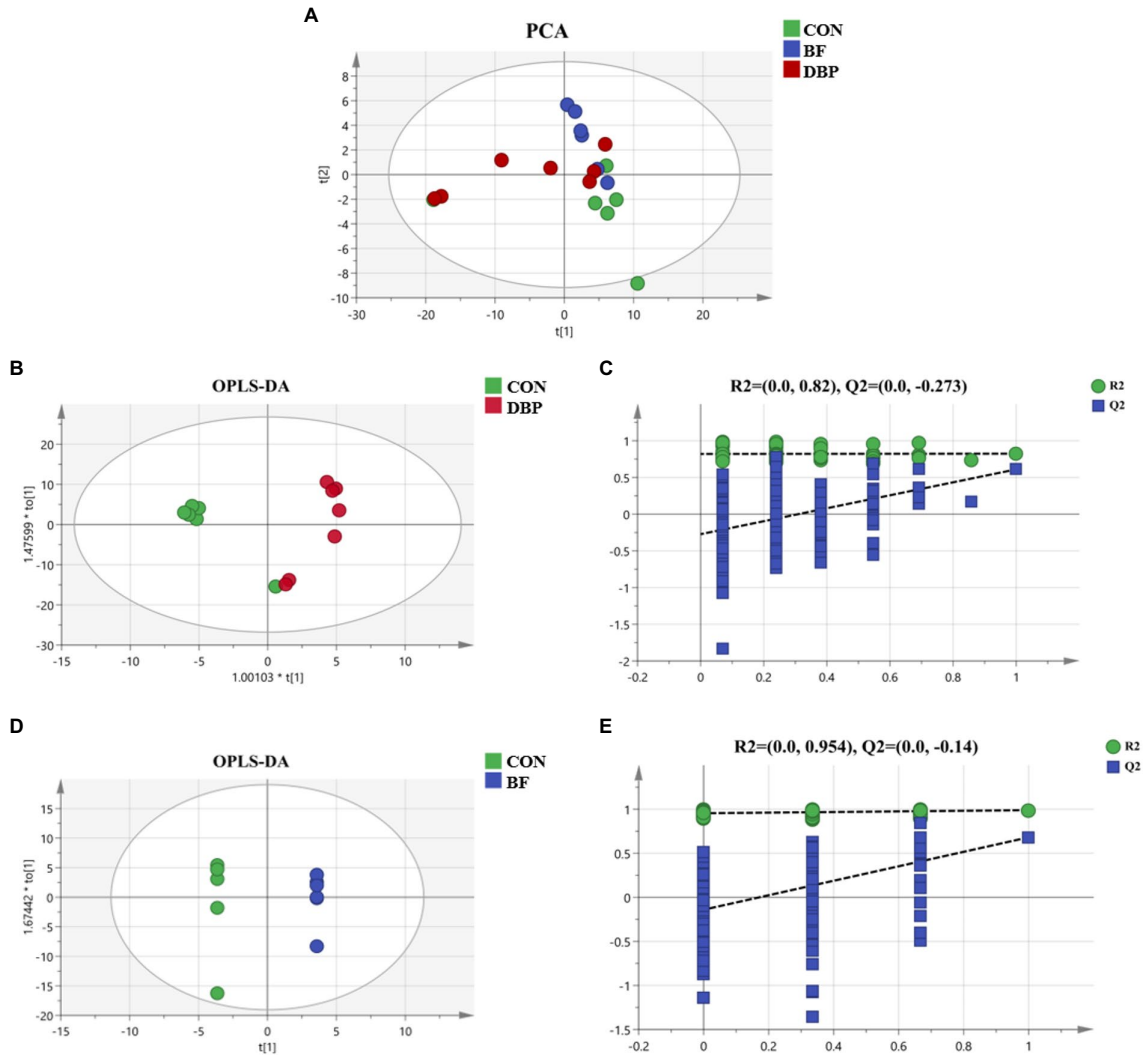
Effects of defatted black soldier fly larvae protein (DBP) and black soldier fly larvae fat (BF) on serum metabolites in dogs. Principal component analysis (PCA) of serum metabolites among the three groups **(A)**. Orthogonal partial least squares discriminant analysis (OPLS-DA) plot of serum metabolites after DBP **(B)**, and BF **(D)**, administration in dogs. Response permutation testing (RPT) derived from the DBP group **(C)**, or the BF group **(E)**, compared with the CON group.

To further explore the influence of DBP and BF on host metabolic profiles in dogs, the serum metabolome among the three groups was monitored. The PCA score plots showed no obvious separation after DBP and BF administration (Figure 1A). Ultimately, the OPLS-DA score plot demonstrated that the DBP and BF groups were separated from the CON group (Figures 1B,D). Additionally, RPT models showed that the models were reliable and had good accuracy and fitness (Figures 1C, E).

Differential serum metabolites were screened out using the standard of VIP (threshold > 1) combined with the *p* value (threshold < 0.05), which was applied to select the significant differential metabolites. We found no differential metabolites between the CON and DBP or BF groups, indicating that feeding 20% DBP or 8% BF had no effect on serum metabolic profiles.

### Effects of DBP and BF on the fecal microbiota in dogs

As demonstrated in Supplementary Figure 1, the Venn diagram revealed 393 shared features between the CON and DBP groups and 864 and 703 in the CON and DBP groups, respectively. The Venn analysis identified 479 shared features between the CON and BF groups and 778 and 617 in the BF and CON groups, respectively. The Venn analysis identified 423 shared features between the DBP and BF groups and 673 and 673 in the DBP and BF groups, respectively. As presented in Supplementary Figure 2A, the alpha diversity, including Observed_species, Shannon, Simpson, Chao1, Goods_coverage, and Pielou_e, of the fecal microbiota showed no difference between the CON and DBP or BF groups (*p* > 0.05). Principal component analysis revealed distinct separation among the three groups (Supplementary Figure 2B), indicating that environment and DBP or BF exhibited no influence on gut microbiota composition or diversity in dogs.

**Figure 2:**
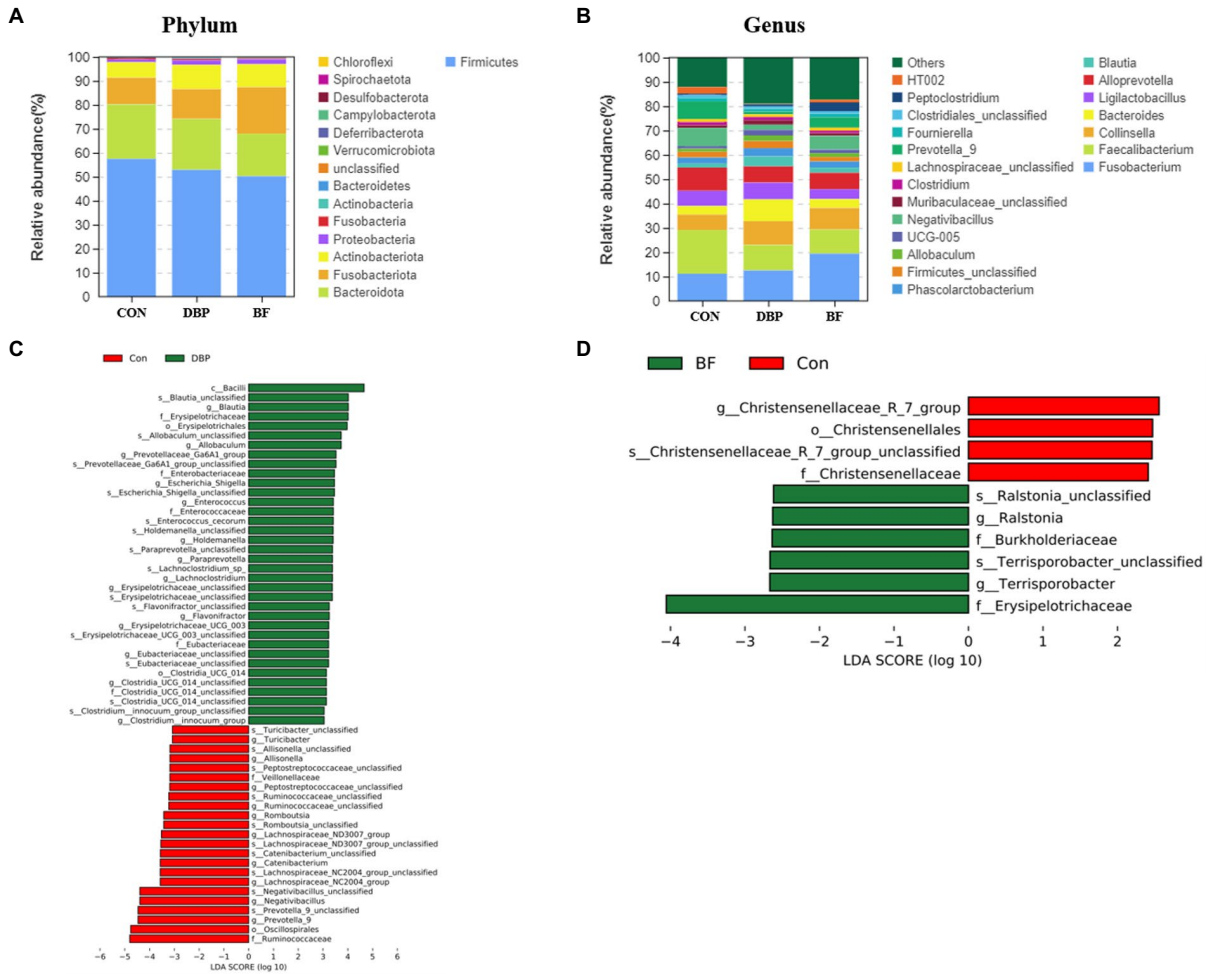
Effects of DBP and BF on gut microbiota and structure in dogs. Histogram of abundance distribution at phylum **(A)**, and genus **(B)**. The LEfSe analysis between the CON group and the DBP **(C)**, or the BF groups **(D)**.

At the phylum level, Firmicutes, Bacteroidetes, Fusobacteria, Actinobacteria, and Proteobacteria were the dominant bacteria and showed no difference among the three groups (Figure 2A). At the genus level, *Fusobacterium*, *Faecalibacterium*, *Collinsella*, *Bacteroides*, *Ligilactobacillus, Alloprevotella*, *Blautia*, and *Phascolarctobacterium* constituted the dominant genera in the top 20 among the three groups (Figure 2B). We identified ASV biomarkers using the LEfSe algorithm. A cladogram representing the fecal microbiota and the predominant species is shown in Figures 2C,D. Compared with the CON group, the DBP group had an elevated relative abundance of *Blautia*, *Allobaculum*, *Prevotellaceae_Ga6A1_group*, *Escherichia_shigella*, *Enterococcus*, *Holdemanella*, *Lachnoclostridium*, *Erysipelotrichaceae_unclassified*, *Flavonifractor*, *Erysipelotrichaceae_UCG_003*, *Clostridia_UCG_014_unclassified*, and *Clostridium_innocuum_group* (Figure 2C). Meanwhile, the BF group was more enriched in *Terrisporobacter* and *Ralstonia* than the CON group (Figure 2D).

### Effects of DBP and BF on the fecal metabolomics in dogs

**Figure 3:**
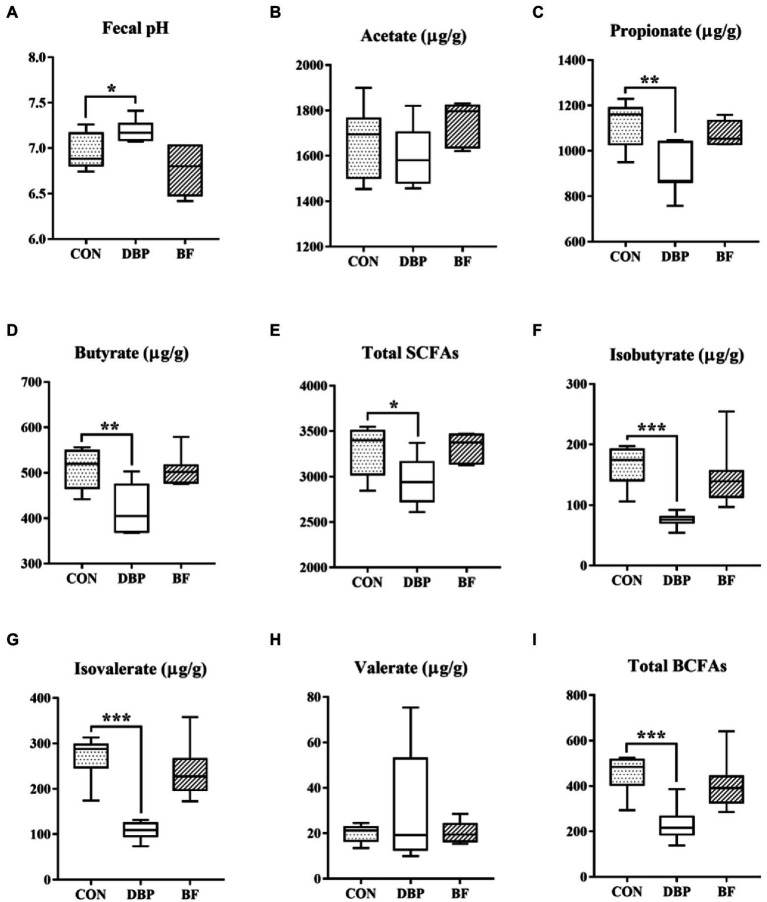
Effects of DBP and BF on fecal pH **(A)**, acetate **(B)**, propionate **(C)**, butyrate **(D)**, total SCFAs **(E)**, isobutyrate **(F)**, isovalerate **(G)**, valerate **(H)**, and total BCFAs **(I)** in dogs. Data are presented as mean ± SE (*n* = 6, 7, or 7). The symbol (^*^) indicates a significant correlation (^*^p < 0.05, ^**^p < 0.01, and ^***^p < 0.001). Total SCFAs = acetate + propionate + butyrate; Total BCFAs = isobutyrate + isovalerate + valerate.

The effects of DBP and BF on fecal fermentation metabolites are shown in Figure 3. Fecal pH was markedly elevated (*p* < 0.05), and propionate, butyrate, total SCFAs, isobutyrate, isovalerate, and total BCFAs were significantly lowered in the DBP group compared to in the CON group (*p* < 0.05), while all the SCFAs and BCFAs showed no difference between the BF and CON groups (*p* > 0.05).

**Figure 4:**
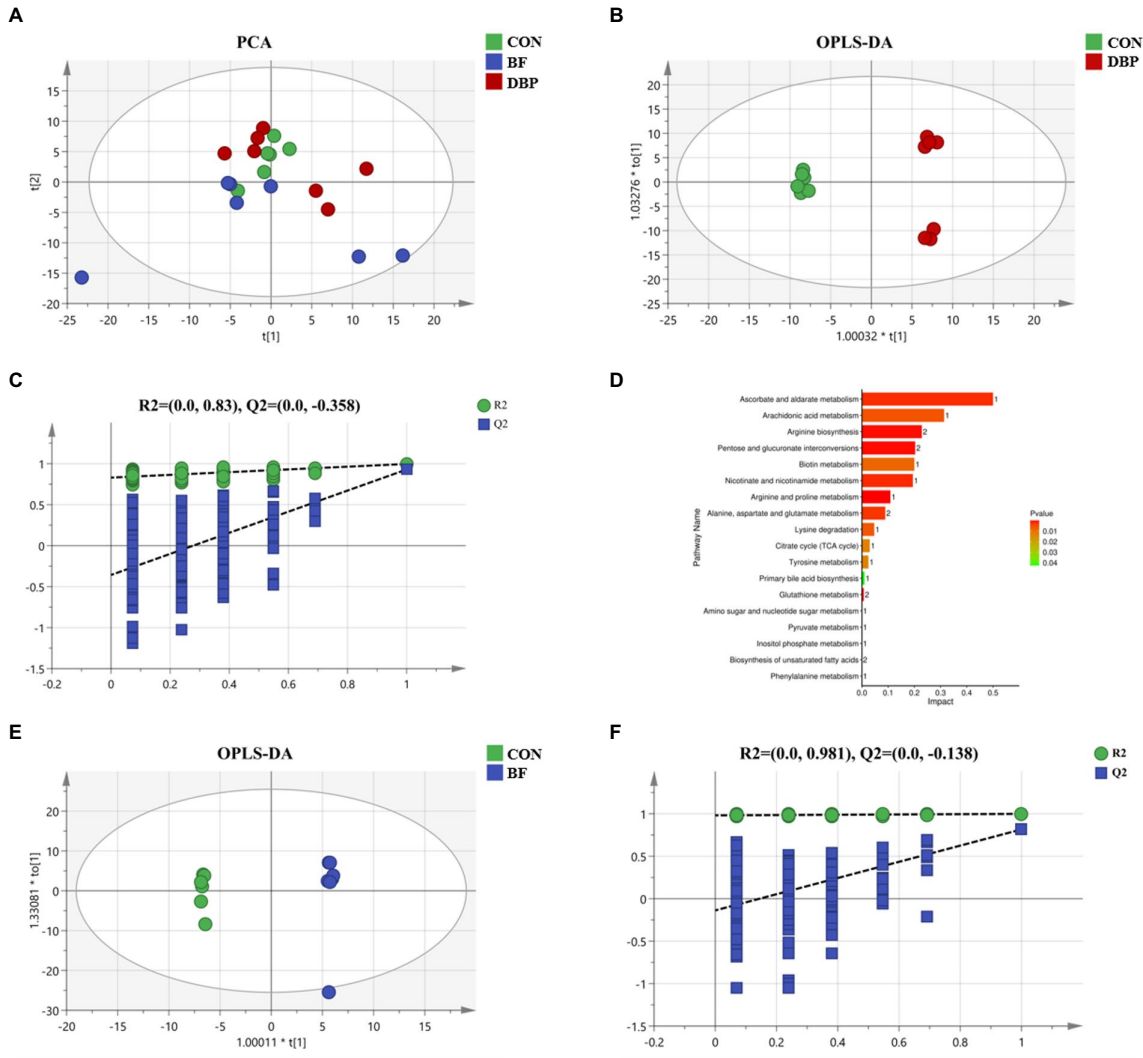
Effects of DBP and BF on fecal metabolites in dogs. PCA of fecal metabolites among the three groups **(A)**. OPLS-DA plot of fecal metabolites between the CON group and DBP group **(B)**, or the BF group **(E)**, in dogs. RPT between the CON group and the DBP group **(C)**, or the BF group **(F)**, in dogs. KEGG metabolic pathways enrichment analysis based on differential fecal metabolites after DBP treatment compared with group **(D)**.

To further investigate the effects of DBP and BF on intestinal microbiota, untargeted metabolomics techniques were used to analyze the contents of metabolites in feces. The PCA score plots showed obvious separation after DBP and BF administration (Figure 4A). In addition, the OPLS-DA model (Figures 4B,E) further separately distinguished the separation in the fecal metabolites between the CON and DBP or BF groups. The quality of the resulting discriminant models between the CON and DBP or BF groups is shown in Figures 4C,F, demonstrating that the models were reliable and predicted.

Differential fecal metabolites were screened out using the standard of VIP > 1 and *p* < 0.05. There were 54 identified potential markers between the CON and DBP groups (Supplementary Table 3), while there were no differential metabolites between the CON and BF groups. In addition, the KEGG analysis further revealed that DBP mainly impacted 18 metabolic pathways, the most dominant of which were arachidonic acid metabolism, arginine biosynthesis, pentose and glucuronate interconversions, biotin metabolism, nicotinate and nicotinamide metabolism, arginine and proline metabolism, alanine, aspartate and glutamate metabolism, and lysine degradation (Figure 4D).

### Effects of DBP and BF on the MetOrigin analysis in dogs

**Figure 5:**
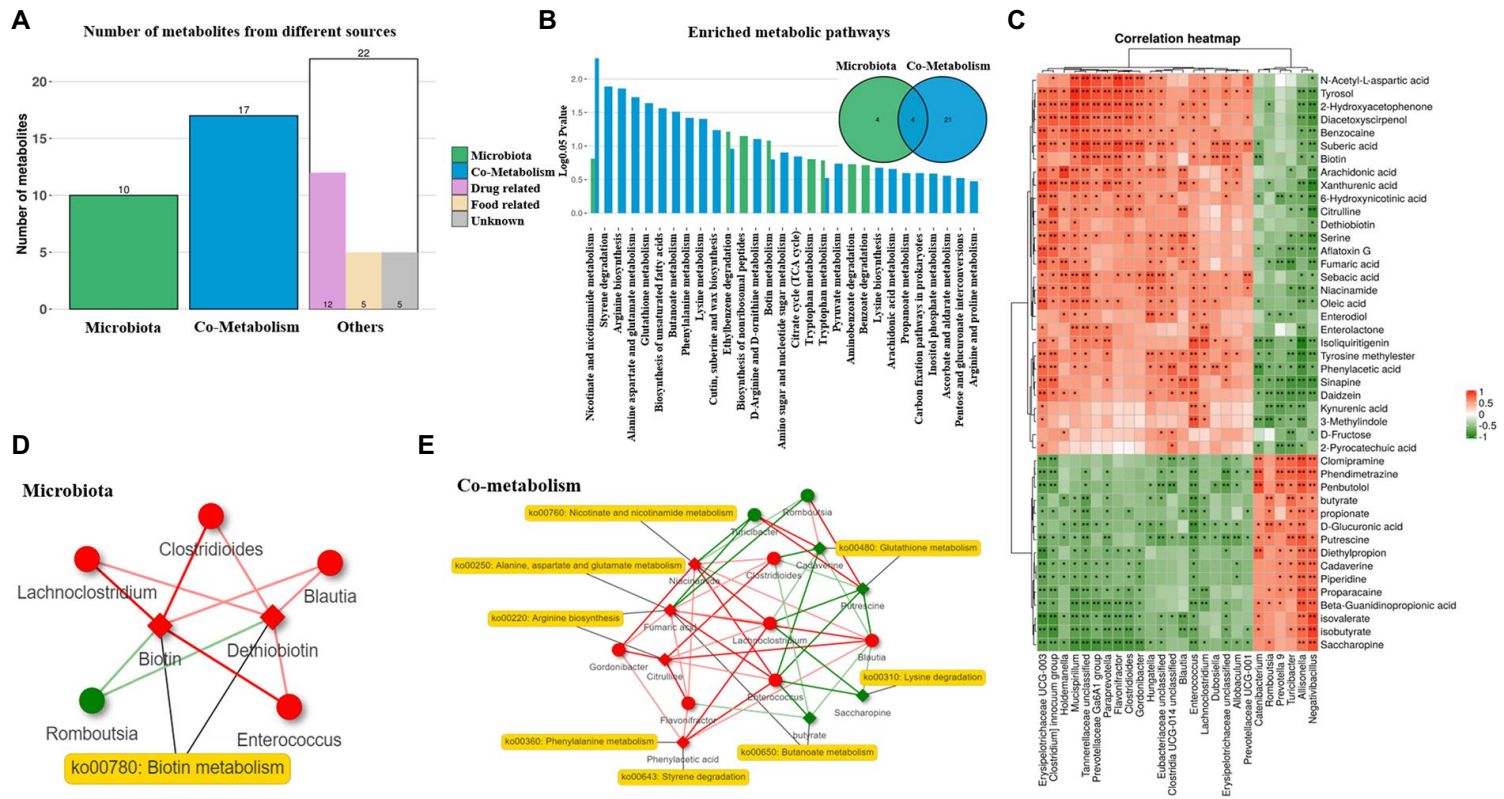
MetOrigin analysis on the differential fecal microbiota and metabolites between the CON and DBP groups. Bar plot of the number of metabolites in different categories **(A)**. Venn diagram and bar plot of the number of enriched metabolic pathways from origin-based MPEA analysis **(B)**. Correlation analysis between the differential fecal microbiota and metabolites using Spearman **(C)**. Network summary of DBP on beagle dogs for microbiota **(D)**, and co-metabolism **(E)**. Diamond and dot shapes indicate correlate metabolites and bacteria, correspondingly. The red/green color of nodes indicates up/down regulation. The red/green lines indicate the positive/negative correlations between microbes and metabolites. The symbol (^*^) indicates a significant correlation (^*^p < 0.05 and ^**^p < 0.01).

To further understand the association between the gut microbiota and metabolic changes, we employed MetOrigin analysis on the differential fecal microbiota and metabolites between the CON and DBP groups. A total of 49 identified metabolites were initially classified into three groups: 10 bacteria-specific metabolites, 17 bacteria-host cometabolites, and 22 others (drug, food, and unknown; Figure 5A). Origin-based metabolic pathway enrichment analysis identified 8 bacteria-specific metabolites and 25 bacteria-host metabolites (Figure 5B). Spearman analysis indicated a strong correlation between the fecal microbiota and metabolites (Figure 5C). The microbiota network of biotin metabolism illustrated that *Clostridioides, Lachnoclostridium*, and *Enterococcus* were positively associated with biotin in the microbiota network (*p* < 0.05), which had been validated by both biological and statistical correlation analysis (Figure 5D). The cometabolism network of nicotinate and nicotinamide metabolism, glutathione metabolism, lysine degradation, arginine biosynthesis, phenylalanine metabolism, styrene degradation, butanoate metabolism, and alanine, aspartate and glutamate metabolism showed that eight metabolites were biologically and statistically associated with eight differential bacteria (*p* < 0.05). Among them, six bacteria (i.e., *Lachnoclostridium*, *Clostridioides*, *Blautia*, *Enterococcus*, *Gordonibacter*, and *Flavonifractor*) and four metabolites (i.e., niacinamide, fumaric acid, citrulline, and phenylacetic acid) were upregulated, while two bacteria (i.e., *Romboutsia* and *Turicibacter*) and another four metabolites (i.e., cadaverine, putrescine, saccharopine, and butyrate) were downregulated by DBF (Figure 5E).

## Discussion

Meat and byproduct meals of poultry, cattle, pig, lamb, and fish are most commonly used as the main protein and fat sources in pet formula (Bosch and Swanson, 2021). These traditional sources of protein and fat are not sufficient to meet the demand for additional feed production; thus, there is an urgent need for alternative protein and fat sources for pet diets (Benzertiha et al., 2020). The amount of protein and fat in insects is comparable to meat (Baiano, 2020), and several novel insect protein and fat sources have been proposed since the last decade (Feng et al., 2018; Hua, 2021). Among them, BSFL has gained substantial attention worldwide as protein (Lalander et al., 2019) and fat (Spranghers et al., 2018; Pinotti et al., 2019; Kim et al., 2020; Li et al., 2022a) substitutes in pet food in recent years due to its economic, nutritional, and environmental advantages (Do et al., 2020). To our knowledge, previous studies have not systematically explored the fungibility of the protein or fat in BSFL. Hence, a 65-day randomized controlled-feeding trial among healthy dogs was implemented. We evaluated apparent nutrient digestibility, serum biochemistry, antioxidant and anti-inflammatory properties, and metabolomics, as well as fecal microbiota and metabolic profiles in the DBP and BF groups compared to the CON group.

The apparent nutrient digestibility reflects the degree of absorption and utilization of the dietary nutrient. In poultry research, DBP meals have been assessed as an excellent source of apparent metabolizable energy and ileal amino acid digestibility (Schiavone et al., 2017). A recent report demonstrated that 1% and 2% DBP elevated CP digestibility in beagle dogs (Lei et al., 2019). In contrast, our findings suggested that 20% DBP reduced the apparent CP and OM digestibility, while 8% BF had no effect on apparent nutrient digestibility. Similar to 20% DBP, previous studies in beagle dogs and cats showed that feeding 20% or 5% BSFL also decreased the apparent CP digestibility (Kröger et al., 2020; Do et al., 2022). Moreover, several studies of BSFL in economic animals (Cullere et al., 2016; Hartinger et al., 2021) and cricket meal in beagle dogs (Kilburn et al., 2020) yielded similar results. Thus, we speculate that the reduction in apparent CP and OM digestibility may be related to the 4.65 ~ 6.43% concentration of chitin in the BSFL (Gariglio et al., 2019; Caimi et al., 2020). Chitin, a linear polymer of β-(1–4) N-acetyl-D-glucosamine units, has high molecular weight, poor water solubility, and protein-binding activity, which makes it difficult to be digested by monogastric animals, has an anti-nutritional effect and has a negative effect on protein digestibility (Longvah et al., 2011). Taking into account the increased proportion of chitin caused by the defatted process, about 5.0 ~ 7.21% (Schiavone et al., 2017; Traksele et al., 2021), the anti-nutritional effect of chitin could explain the reduction of apparent CP and OM digestibility. However, the underlying mechanisms remain to be elucidated (Penazzi et al., 2021).

The serum biochemistry, antioxidant, and anti-inflammatory properties and metabolomics were analyzed to confirm the safety of feeding DBP and BF. Our results revealed that the serum biochemistry and metabolomics were within the normal range with no difference among the three groups, illustrating that neither 20% DBP nor 8% BF affected the heath of all dogs in the experiment. A study by Kröger et al. (2020) reached a similar conclusion that 20% BSFL had no effect on serum biochemistry in adult dogs, and a cricket meal evaluation on beagle dogs also found that all blood values remained within desired reference intervals (Kilburn et al., 2020). In weaning piglets, 2% BF regulated serum GLO, TP, and TG, decreased IFN-γ levels and increased IL-10 and IgA levels (Yu et al., 2020). Likewise, in this study, a lower level but no significant difference in serum IFN-γ level was observed in the BF group, indicating that the anti-inflammatory potential of BF may be due to the anti-inflammatory effects of lauric acid and linoleic acid in BSFL (Kim et al., 2020; Darwish et al., 2021). In addition, a study on beagle dogs found a decreasing serum TNF-α level and an increasing GSH-Px level that linearly altered with increasing DBP (0, 1, and 2%; Lei et al., 2019), and beagle dogs treated with house flies revealed a lower serum MDA level (Hong et al., 2020). However, no difference was observed between the CON and DBP groups in our study, indicating that a high proportion of DBP (20%) did not exert antioxidant or anti-inflammatory effects.

Insects are known to contain nondigestible components that are important fermentable substrates for the colonic microbiota (Bosch et al., 2016). Thus, we further explored the changes in gut microbial composition of the DBP and BF groups. No differences were noted in the α- and β-diversity of the fecal microbiota among the three groups, indicating that 20% DBP and 8% BF had no obvious effect on the gut microbial richness or diversity in adult dogs. Consistent with previous studies (Pilla and Suchodolski, 2020; Yang K. et al., 2022a,b), Firmicutes, Bacteroidetes, Fusobacteria, Actinobacteria, and Proteobacteria were the dominant bacterial phyla. Moreover, *Fusobacterium*, *Faecalibacterium*, *Collinsella*, and *Bacteroides* were the dominant bacterial genera among the three groups and showed no difference between the CON and DBP or BF groups. Upon further analysis of bacterial genera, we found that 20% DBP decreased the relative abundance of SCFA-producing bacteria, including *Prevotella_9* (Li et al., 2022b), *Lachnospiraceae_NC2004_group* (Egerton et al., 2022), *Catenibacterium* (Liu et al., 2021), *Allisonella* (Zhao et al., 2021), *Turicibacter*, and *Romboutsia* (Li et al., 2021), and a previous study confirmed that *Flavonifractor* (Hong et al., 2021), *Blautia*, and *Enterococcus* (Kellingray et al., 2018) acted as inhibitors of butyrate, resulting in a reduction in butyrate in feces, thereby increasing fecal pH. SCFAs are produced by the microbial fermentation of undigestible carbohydrates (Kawauchi et al., 2011). Numerous studies have demonstrated the beneficial roles of SCFAs, including maintaining host immunity and nutritional metabolism (Song et al., 2021) and positively affecting the regulation of inflammation and intestinal barrier function (Liu et al., 2021). In addition, we found that 20% DBP increased the relative abundance of *Blautia,* which exhibited a negative correlation with the levels of fecal SCFAs (Pérez-Burillo et al., 2019; Lin et al., 2021). Meanwhile, increasing *Escherichia_shigella* (Cattaneo et al., 2017; Yang L. et al., 2022), *Enterococcus*, *Holdemanella* (Xu et al., 2021), *Lachnoclostridium* (Chen et al., 2021), and *Flavonifractor* (Straub et al., 2021) in the DBP group have potentially negative consequences for gut health (Sekirov et al., 2010). Unlike our results, a study of cricket on the gut microbiota in beagle dogs demonstrated that cricket decreased the abundance of *Faecalibacterium* and *Bacteroides* (Jarett et al., 2019). It is known that the gut microbiota generates BCFAs as a result of proteolysis of undigested proteins and deamination of branched-chain amino acids (Badri et al., 2021). We found that the microbial alterations obviously reduced the concentrations of isobutyrate, isovalerate, and total BCFAs in the DBP group. One of the key reasons for this was that the high proportion of chitin constrained the utilizability of protein (Gariglio et al., 2019), thereby affecting BCFA production. In brief, long-term feeding with 20% DBP may have adverse effects on canine health. The present study also found that the BF group recruited more *Terrisporobacter,* which played a key role as a beneficial intestinal bacterium (Lin et al., 2020), indicating that 8% BF may act as an anti-inflammatory (a lower level of serum IFN-γ) effect by enhancing the production of beneficial bacteria.

Gut microbial metabolites are closely associated with nutritional status, metabolism, and stress response (Yang et al., 2021). Therefore, untargeted metabolomics was performed to determine the effects of DBP and BF on fecal metabolic profiles. The PCA and OPLS-DA plots showed that the DBP group rather than the BF group had a distinct separation of fecal metabolites from the CON group. To further confirm the biological relationships of metabolites to gut microbiota, MetOrigin, an interactive web server that discriminates metabolites originating from the microbiome, was applied to explore their relationship (Yu et al., 2022). From the outcome of the microbiota network *via* MetOrigin analysis, we found that feeding 20% DBP increased the relative abundances of *Lachnoclostridium*, *Clostridioides*, and *Enterococcus*, which metabolized desthiobiotin to produce biotin by secreting biotin synthase, in turn modulating the biotin metabolic pathway. Biotin, a cofactor of intermediary metabolism, is covalently attached to enzymes (Sirithanakorn and Cronan, 2021), which may have therapeutic potential for patients with inflammatory bowel disease (Skupsky et al., 2020). Moreover, in the cometabolism network, we found that *Lachnoclostridium*, *Blautia*, *Enterococcus*, and *Clostridioides* upregulated niacinamide, phenylalanine acid, fumaric acid, and citrulline and downregulated cadavrine, putrescine, saccharopine, and butyrate. Among them, glutathione metabolism and nicotinate and nicotinamide metabolism participate in antioxidant activity (Seo et al., 2018) and protect against aging (Si et al., 2019), respectively, which may benefit gut health in dogs. Moreover, 20% DBP increased the relative abundances of *Flavonifractor*, *Blautia*, and *Enterococcus*, which inhibited the secretion of acetoacetate CoA-transferase, thereby reducing the production of butyrate (Kellingray et al., 2018; Hong et al., 2021). This further verified our conclusion.

Overall, our findings confirmed that 20% DBP restrained the apparent CP and OM digestibility, thereby affecting hindgut microbial metabolism, while 8% BF had no negative effect on canine gut health. Specifically, taking into account the increased proportion of chitin caused by the defatted process, continued efforts are warranted in understanding the chitin effects. We suggest that adding high-quality chitinase to dog food may be an effective way to improve the apparent nutrient digestibility of DBP.

## Conclusion

The current findings suggested that neither 20% DBP nor 8% BF affected the body condition of all dogs in this experiment. 20% DBP had decreasing apparent CP and OM digestibility on day 65, while 8% BF had no effect on apparent nutrient digestibility. Furthermore, the DBP group had decreasing fecal propionate, butyrate, total SCFAs, isobutyrate, isovalerate, and total BCFAs and increased fecal pH. Nevertheless, there was no difference in SCFAs and BCFAs between the CON and BF groups. The fecal microbiota revealed that *Lachnoclostridium*, *Clostridioides*, *Blautia*, and *Enterococcus* were enriched in the DBP group, and *Terrisporobacter* and *Ralstonia* were enriched in the BF group. The fecal metabolome further showed that the DBP group significantly influenced 18 metabolic pathways. Additionally, MetOrigin analysis between the CON and DBP groups found that *Lachnoclostridium*, *Clostridioides*, and *Enterococcus* were positively associated with biotin. In addition, *Lachnoclostridium*, *Clostridioides*, *Blautia*, and *Enterococcus* were positively associated with niacinamide, phenylalanine acid, fumaric acid, and citrulline and negatively associated with cadavrine, putrescine, saccharopine, and butyrate. Overall, 20% DBP restrained the apparent CP and OM digestibility, thereby affecting hindgut microbial metabolism. In contrast, 8% BF in the dog diet showed no adverse effects on body condition, apparent nutrient digestibility, fecal microbiota, or metabolic profiles. Our findings are conducive to opening a new avenue for the exploitation of DBP and BF as protein and fat resources in dog food.

## Data availability statement

The 16S rRNA data presented in the study are deposited in the NCBI repository, accession number: https://www.ncbi.nlm.nih.gov/, PRJNA865813. The untargeted metabolomic data presented in the study are deposited in the EMBL-EBI MetaboLights repository with the identifier MTBLS6097 (serum metabolomics) and MTBLS6102 (fecal metabolomics), accession numbers corresponding to the following: https://www.ebi.ac.uk/metabolights/MTBLS6097 and https://www.ebi.ac.uk/metabolights/MTBLS6102.

## Ethics statement

The animal study was reviewed and approved by Experimental Animal Ethics Committee of South China Agricultural University (approval code 2021E028).

## Author contributions

SJ and LZ designed the study and performed the experiments. SJ detected the samples, analyzed the data, and prepared the manuscript. ND, KY and ZX carried out the assays described in the study. MH, ZdZ and ZhZ contributed to the data analysis. KY and BD revised the manuscript. JD and BD provided the funding and resources. All authors contributed to the article and approved the submitted version.

## Funding

This project was supported by National Natural Science Foundation of China (Grant Nos. 32172744, 31790411, and 32002186), National Key R&D Program of China (Grant No. 2021YFD1300400), Natural Science Foundation of Guangdong Province (Grant No. 2020A1515010322), Guangzhou Basic and Applied Basic Research Foundation (Grant No. 202102020850), Start-up Research Project of Maoming Laboratory (Grant No. 2021TDQD002), and Science and Technology Planning Project of Guangdong Province (2021B1212060001).

## Conflict of interest

LZ is employed by Guangzhou Qingke Biotechnology Co., Ltd., ND is employed by Guangzhou Customs Technology Center, and MH, ZdZ, and ZhZ are employed by Guangzhou GeneralPharmaceutical Research Institute Co., Ltd. (National CanineLaboratory Animal Resources Center).

The remaining authors declare that the research was conducted in the absence of any commercial or financial relationships that could be construed as a potential conflict of interest.

## Publisher’s note

All claims expressed in this article are solely those of the authors and do not necessarily represent those of their affiliated organizations, or those of the publisher, the editors and the reviewers. Any product that may be evaluated in this article, or claim that may be made by its manufacturer, is not guaranteed or endorsed by the publisher.

## Footnotes

^1^ http://huttenhower.sph.harvard.edu/lefse/

^2^ https://www.metaboanalyst.ca
