# Supplementary material for: Effects of black soldier fly larvae as protein or fat sources on apparent nutrient digestibility, fecal microbiota, and metabolic profiles in beagle dogs
Source: Front Microbiol. 2022 Nov 25;13:1044986. doi: 10.3389/fmicb.2022.1044986 (PMC9733673; doi:10.3389/fmicb.2022.1044986)
Supplement: Supplementary file 1 [file Data_Sheet_1.docx]

Supplementary Material

**
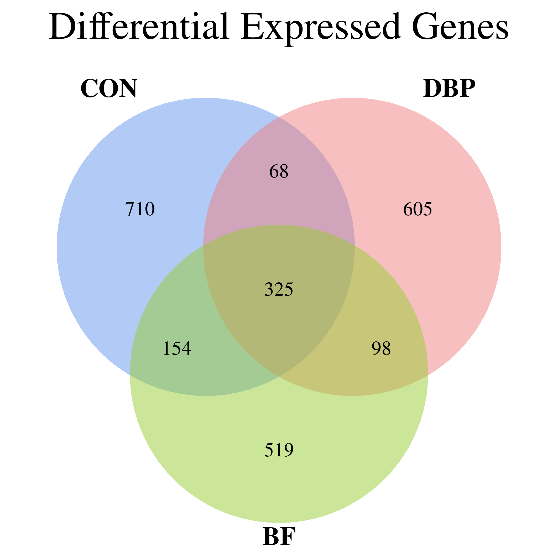
**

**Supplementary Figure** | Venn among the CON and DBP and BF groups.


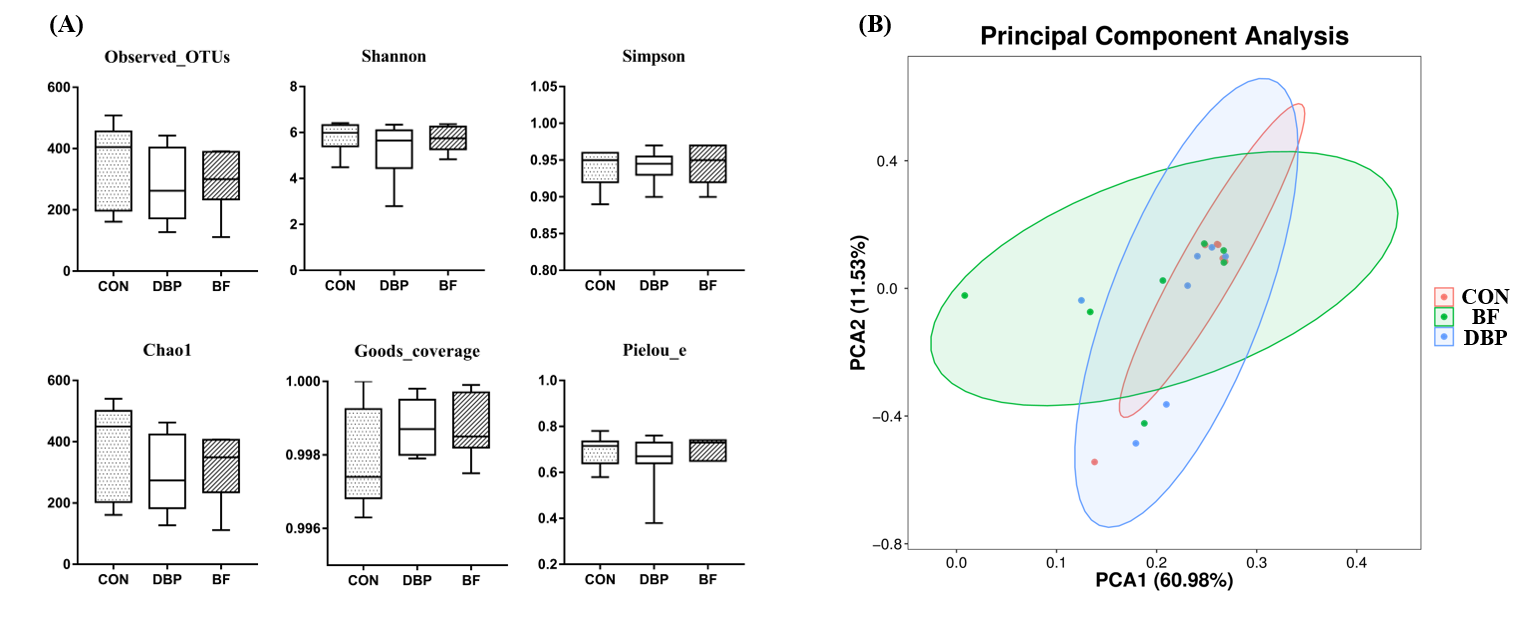


**Supplementary Figure 2** | Effects of DBP and BF on alpha-diversity analysis (Observed_OTUs, Shannon, Simpson, Chao1, Goods_coverage, and Pielou_e) **(A)** and principal component analysis **(B)** of fecal microbiota in dogs.

**Supplementary Table 1** | Proximate analysis of chicken meal and DBP.

| **Items^1^** | **Chicken meal** | **DBP** |
| --- | --- | --- |
| Proximate analysis | as-dry matter basis | |
| DM (%) | 91.52 | 93.64 |
| OM (%) | 86.19 | 89.86 |
| CP (%) | 68.34 | 58.84 |
| EE (%) | 10.51 | 5.29 |
| GE (MJ/kg) | 21.2 | 21.5 |
| Moisture (%) | 8.47 | 6.17 |
| Ash (%) | 13.82 | 10.28 |
| Chitin (%) | -- | 5.0~7.21% |

*^1^ DBP: Defatted black soldier fly larvae protein group; DM: Dry matter; OM: Organic matter; CP: Crude protein; EE: Ether extract; GE: Gross energy.*

*2 The chitin content in black soldier fly was estimated according to* [*Traksele*](https://www.webofscience.com/wos/alldb/general-summary?queryJson=%5B%7B%22rowBoolean%22:null,%22rowField%22:%22AU%22,%22rowText%22:%22Traksele,%20Lina%22%7D%5D&eventMode=oneClickSearch) *et al. and* [*Schiavone*](https://www.webofscience.com/wos/alldb/general-summary?queryJson=%5B%7B%22rowField%22:%22AU%22,%22rowText%22:%22Schiavone,%20A%22%7D%5D&eventMode=oneClickSearch) *et al.*  (Schiavone et al., 2017; Traksele et al., 2021)*.*

**Supplementary Table 2** | Effects of DBP and BF on serum biochemistry parameters in dogs.

| **Items^1^** | **Normal range^2^** | **CON** | **DBP** | **BF** | ***P*-value (CON/DBP)** | ***P*-value (CON/BF)** |
| --- | --- | --- | --- | --- | --- | --- |
| ALB (g/L) | 23.0~40.0 | 34.62±2.04 | 35.17±1.64 | 35.13±2.66 | 0.619 | 0.703 |
| TP (g/L) | 52.0~82.0 | 67.38±2.77 | 66.52±2.76 | 68.93±5.78 | 0.599 | 0.546 |
| GLO (g/L) | 23.0~45.0 | 32.77±1.87 | 31.37±2.94 | 33.80±5.08 | 0.349 | 0.648 |
| ALB/GLO | 0.80~2.00 | 1.06±0.09 | 1.13±0.12 | 1.06±0.17 | 0.281 | 1.000 |
| AST (U/L) | 0~50 | 38.67±5.50 | 38.00±5.40 | 39.14±7.88 | 0.837 | 0.901 |
| ALT (U/L) | 5~125 | 41.5±4.46 | 39.17±7.63 | 36.71±8.96 | 0.536 | 0.244 |
| AMY (U/L) | 400~1500 | 842.67±125.64 | 799.33±119.62 | 886.00±645.95 | 0.554 | 0.867 |
| CK (U/L) | 10~200 | 172.00±67.98 | 151.83±33.84 | 188.14±49.21 | 0.535 | 0.641 |
| CRE (μmol/L) | 44.0~159.0 | 79.73±6.99 | 77.92±15.52 | 75.7±9.83 | 0.801 | 0.408 |
| BUN (mmol/L) | 2.50~9.60 | 5.45±0.46 | 5.46±0.65 | 5.58±0.74 | 0.992 | 0.714 |
| BUN/CRE | 16.00~218.00 | 68.86±9.26 | 71.74±12.26 | 74.48±10.96 | 0.657 | 0.338 |
| GLU (mmol/L) | 4.11~7.94 | 4.41±1.13 | 4.30±0.44 | 4.48±0.36 | 0.834 | 0.888 |
| Ca (mmol/L) | 1.0~1.6 | 2.51±0.07 | 2.46±0.06 | 2.51±0.12 | 0.245 | 0.940 |
| IP (mmol/L) | 0.8~1.2 | 1.18±0.13 | 1.06±0.23 | 1.00±0.20 | 0.281 | 0.074 |

*^1^ CON: Basal diet group; DBP: Defatted black soldier fly larvae protein group; BF: Black soldier fly larvae fat group; ALB: Albumin; TP: Total protein; GLO: Globulin; ALB/GLO: Albumin/globulin; AST: Aspartate aminotransferase; ALT: Alanine transaminase; AMY: Amylase; CK: Creatine kinase; CRE: Creatinine; BUN: Urea nitrogen; GLU: Glucose; Ca: Calcium; IP: Inorganic phosphorus.*

*^2^ The normal range of all serum biochemistry parameters was described in a previous study* (Yang et al., 2022)*.*

**Supplementary Table 3** | Significant metabolites between the CON and DBP groups.

| **Metabolites** | **VIP** | ***P*-value** | **Fold change** | **Trend**  **(CON/DBP)** |
| --- | --- | --- | --- | --- |
| Sinapine | 1.75795 | 1.80E-13 | 0.000922 | down |
| Phenylacetic acid | 1.81327 | 4.04E-12 | 0.008137 | down |
| Tyrosine methylester | 1.78479 | 1.43E-09 | 0.093601 | down |
| Aflatoxin G | 1.89863 | 1.02E-08 | 0.16597 | down |
| 2-Methoxy-3-(1-methylpropyl) pyrazine | 1.91472 | 1.18E-06 | 0.23606 | down |
| Proparacaine | 1.92412 | 1.66E-06 | 3.429 | up |
| Daidzein | 1.74624 | 2.42E-06 | 0.094885 | down |
| 6-Hydroxynicotinic acid | 1.93635 | 5.19E-06 | 0.20587 | down |
| Xanthurenic acid | 1.80808 | 7.16E-06 | 0.28212 | down |
| Niacinamide | 1.61818 | 1.35E-05 | 0.21131 | down |
| Diethylpropion | 1.79207 | 1.41E-05 | 2.2636 | up |
| Piperidine | 1.56784 | 1.96E-05 | 13.052 | up |
| Cadaverine | 1.56725 | 2.03E-05 | 13.382 | up |
| Methyl 1-methoxy-1H-indole-3-carboxylate | 1.25342 | 2.85E-05 | 0.28704 | down |
| 2'-Hydroxyacetophenone | 1.65414 | 2.97E-05 | 0.16266 | down |
| Penbutolol | 1.91136 | 3.28E-05 | 2.455 | up |
| Tyrosol | 1.64037 | 3.80E-05 | 0.15843 | down |
| Beta-Guanidinopropionic acid | 1.70123 | 4.41E-05 | 3.6993 | up |
| p-Methylhippuric acid | 1.81043 | 5.55E-05 | 2.1782 | up |
| Isoliquiritigenin | 1.66319 | 7.55E-05 | 0.15566 | down |
| Phendimetrazine | 1.87166 | 0.000127 | 2.2024 | up |
| Serine | 1.14884 | 0.000183 | 0.35942 | down |
| Oleic acid | 1.59171 | 0.000209 | 0.30961 | down |
| Benzocaine | 1.66415 | 0.000267 | 0.40939 | down |
| Putrescine | 1.79457 | 0.000371 | 2.6513 | up |
| D-Xylose | 1.02513 | 0.00038 | 0.49958 | down |
| Diacetoxyscirpenol | 1.61042 | 0.000519 | 0.36221 | down |
| Indole-3-methyl acetate | 1.05805 | 0.000537 | 0.31481 | down |
| Sebacic acid | 1.21358 | 0.000593 | 0.431 | down |
| Suberic acid | 1.69323 | 0.000693 | 0.42097 | down |
| Citrulline | 1.22173 | 0.000698 | 0.17528 | down |
| Acetylglycine | 1.25988 | 0.000714 | 0.14982 | down |
| D-Fructose | 1.17292 | 0.000758 | 0.39675 | down |
| Fumaric acid | 1.27859 | 0.000782 | 0.44373 | down |
| Alanyl-Phenylalanine | 1.04926 | 0.001243 | 0.18832 | down |
| 6-Methylnicotinamide | 1.42254 | 0.001331 | 0.49538 | down |
| Monobutylphthalate | 1.08174 | 0.001433 | 0.39593 | down |
| Clomipramine | 1.87023 | 0.001454 | 2.2743 | up |
| Saccharopine | 1.66945 | 0.001568 | 3.0566 | up |
| Dethiobiotin | 1.26688 | 0.001602 | 0.43063 | down |
| Sinapic acid | 1.07725 | 0.002012 | 0.32802 | down |
| Kynurenic acid | 1.06621 | 0.002282 | 0.47691 | down |
| D-Glucuronic acid | 1.51943 | 0.002825 | 2.6166 | up |
| Arachidonic acid | 1.30729 | 0.003232 | 0.3728 | down |
| Taurochenodesoxycholic acid | 1.08443 | 0.005308 | 0.33343 | down |
| Aminosalicylic acid | 1.00487 | 0.006873 | 0.47395 | down |
| 3-Methylindole | 0.888361 | 0.006886 | 0.35469 | down |
| Biotin | 1.38246 | 0.007332 | 0.31306 | down |
| N-Acetyl-L-aspartic acid | 1.25387 | 0.007509 | 0.46188 | down |
| Enterodiol | 1.26624 | 0.007945 | 0.3106 | down |
| 2-Pyrocatechuic acid | 0.843046 | 0.010786 | 0.075006 | down |
| 3,3-Dimethylglutaric acid | 1.19394 | 0.013344 | 0.37895 | down |
| Enterolactone | 1.30094 | 0.013799 | 0.25941 | down |
| gamma-Asarone | 0.814594 | 0.01457 | 0.36264 | down |

*CON: Basal diet group; DBP: Defatted black soldier fly larvae protein group; BF: Black soldier fly larvae fat group.*

**References**

Schiavone, A., De Marco, M., Martínez, S., Dabbou, S., Renna, M., and Madrid, J., et al. (2017). Nutritional value of a partially defatted and a highly defatted black soldier fly larvae (Hermetia illucens L.) meal for broiler chickens: Apparent nutrient digestibility, apparent metabolizable energy and apparent ileal amino acid digestibility. *Journal of Animal Science and Biotechnology*. 8(1), 51. doi: 10.1186/s40104-017-0181-5

Traksele, L., Speiciene, V., Smicius, R., Alencikiene, G., Salaseviciene, A., and Garmiene, G., et al. (2021). Investigation of in vitro and in vivo digestibility of black soldier fly (Hermetia illucens L.) larvae protein. *J. Funct. Foods*. 79, 104402. doi: 10.1016/j.jff.2021.104402

Yang, K., Jian, S., Wen, C., Guo, D., Liao, P., and Wen, J., et al. (2022). Gallnut tannic acid exerts anti-stress effects on stress-induced inflammatory response, dysbiotic gut microbiota, and alterations of serum metabolic profile in beagle dogs. *Front Nutr*. 9, 847966. doi: 10.3389/fnut.2022.847966
